# Building clinically meaningful and implementable interventions for persons with primary progressive aphasia and their communication partners: Lessons from the Communication Bridge Trials

**DOI:** 10.1002/alz70858_100321

**Published:** 2025-12-25

**Authors:** Emily J Rogalski, Ollie Fegter, Alfred Rademaker, Stella Wang, Rhiana Schafer, Matthew Bona, Angela C. Roberts

**Affiliations:** ^1^ Healthy Aging & Alzheimer's Research Care (HAARC) Center, Healthy Aging & Alzheimer's Research Care (HAARC) Center, The University of Chicago, Chicago, IL, USA; ^2^ Northwestern University Feinberg School of Medicine, Chicago, IL, USA; ^3^ University of Chicago, Chicago, IL, USA; ^4^ Northwestern University, Chicago, IL, USA; ^5^ The University of Chicago, Chicago, IL, USA; ^6^ Healthy Aging & Alzheimer's Research Care (HAARC) Center, University of Chicago, Chicago, IL, USA; ^7^ Northwestern University, Evanston, IL, USA; ^8^ Western University, London, ON, Canada

## Abstract

**Background:**

Primary progressive aphasia (PPA) is a currently incurable language‐based neurodegenerative dementia syndrome, which negatively impacts communication and quality of life. The Communication Bridge‐2 (CB2) trial was the first international telemedicine speech‐language (RCT) to show efficacy for persons with PPA. Despite the promising results of the CB2 trial, translating these findings into routine clinical practice poses an urgent and significant challenge. Primary, secondary, and stakeholder data were used to assess readiness for pragmatic randomized controlled trials and implementation.

**Method:**

CB2 was an internationally enrolling, single enrollment site, Phase 2, Stage 2, parallel‐group, active control, behavioral RCT delivered via video‐chat to individuals with PPA and their communication partners. Participation was ∼12 months. A custom web application supported CB2 delivery. Post‐study interviews (PSI) conducted with the dyad and a research clinician blinded to intervention arm captured participant experiences and informed Readiness Assessment for Pragmatic Trials (RAPT) ratings. Outcomes were compared between arms qualitatively and using the cumulative logit link in which groups were compared using proportional odds at each evaluation.

**Result:**

Ninety‐five PPA participant dyads (mean enrollment age of PPA participants: 67.1 years, 48% female) were randomized (*n* = 4 countries). Dropout was <10%. PSI assessed the dyad‐perceived impact of the intervention on everyday life and provided data regarding the feasibility and acceptability of the intervention, aligned with the RAPT model. Reports were positive for both arms; however, responses favored the Experimental arm for positive changes in communication in daily activities (Odds Ratio (OR):2.9, *p* = 0.073), positive changes in emotional wellbeing (OR:1.4, *p* = 0.47), overall helpfulness of the speech therapy intervention (OR:4.4, *p* = 0.083), and reached between group significance in meeting overall expectations of the intervention (OR:5.4, *p* = 0.005). Participants in both groups showed high usage and feasibility in using the home exercise app.

**Conclusion:**

Improving outcomes for PPA, Alzheimer's disease, and Related Dementias (ADRD) requires ensuring that evidence‐based interventions are accessible, scalable, and integrated into real‐world settings. Evidence from CB2 and stakeholder feedback indicates the intervention is on a strong path towards implementation. These data provide a model for developing future ADRD non‐pharmacologic interventions.

